# ReFOLD: a server for the refinement of 3D protein models guided by accurate quality estimates

**DOI:** 10.1093/nar/gkx249

**Published:** 2017-04-10

**Authors:** Ahmad N. Shuid, Robert Kempster, Liam J. McGuffin

**Affiliations:** 1 School of Biological Sciences, University of Reading, Whiteknights, Reading RG6 6AS, UK; 2 Lancaster Environment Centre, Lancaster University, LA1 1YQ, UK

## Abstract

ReFOLD is a novel hybrid refinement server with integrated high performance global and local Accuracy Self Estimates (ASEs). The server attempts to identify and to fix likely errors in user supplied 3D models of proteins via successive rounds of refinement. The server is unique in providing output for multiple alternative refined models in a way that allows users to quickly visualize the key residue locations, which are likely to have been improved. This is important, as global refinement of a full chain model may not always be possible, whereas local regions, or individual domains, can often be much improved. Thus, users may easily compare the specific regions of the alternative refined models in which they are most interested e.g. key interaction sites or domains. ReFOLD was used to generate hundreds of alternative refined models for the CASP12 experiment, boosting our group's performance in the main tertiary structure prediction category. Our successful refinement of initial server models combined with our built-in ASEs were instrumental to our second place ranking on Template Based Modeling (TBM) and Free Modeling (FM)/TBM targets. The ReFOLD server is freely available at: http://www.reading.ac.uk/bioinf/ReFOLD/.

## INTRODUCTION

The refinement of 3D models of proteins can be thought of as the ‘end game’ of protein structure prediction (1). Subtle improvements in predictive models are often required to move them the ‘last mile’, beyond the template or fragments upon which they are based, so they more accurately reflect the time averaged observed structures. Comparative protein modelling is now routinely used across the life sciences. However, many biological applications of 3D models are critically dependent on high model accuracy, particularly in key regions, such as binding sites for drug discovery (2). Improving the accuracy of comparative models, beyond the information derived from the template, therefore continues to be one of the pressing problems in structural bioinformatics. However, it has proved difficult to develop reliable and practically useful refinement methods, highlighted by the relatively slow progress seen in CASP (Critical Assessment of Techniques for Protein Structure Prediction). Indeed, the CASP assessors have stated that ‘the endgame problem is going to be at least several orders of magnitude harder than the TBM (Template Based Modeling) problem’ (1).

Refinement was introduced as a new category in CASP7 to encourage development. While quality assessment (QA) methods, such as ModFOLD (3), can accurately identify the magnitude of errors in models and locate where those errors are, refinement methods aim to fix the errors in models. Once an atomic model has been obtained, it may be tweaked to idealize bond geometry and to remove unfavorable contacts that may have been introduced by the initial modelling process. Ironically, in the early years of refinement, often most methods made models worse, rather than improving upon them. In recent years, the situation has been improved, however, it is hard to quantify if improvements between CASPs are being made in refinement, as maintaining the level of ‘target difficulty’ is problematic (1).

Refinement methods can be loosely categorized into the automated server-based programs and non-server-based highly CPU intensive programs, referred to as the ‘human’ refinement groups in the CASP experiments as they allow a large degree of manual intervention in their pipelines. The freely available automated server GalaxyRefine, focuses on rebuilding and repacking of the side chains, before performing overall structure relaxation via molecular dynamic simulation (MDS) (4). Additionally, the KoBaMIN server, relies on minimization of a knowledge-based potential of mean force (5). Another successful fully automated method is the 3Drefine method (6,7), which works in a two-step process involving optimization of the hydrogen bonding network and composite physics and knowledge-based force fields to give atomic-level energy minimization using the MESHI molecular modelling framework (8). The advantage of such automated approaches is that they are easily distributed, accessible and user-friendly. However, successes in improving protein models via automated approaches remains relatively low, with the most successful servers making relatively conservative changes. In contrast, the less well automated approaches, which often make more bold changes to models, have arguably achieved better results.

The human refinement methods commonly use physics based approaches that select structures from Molecular Dynamics (MD)-based ensembles followed by structural averaging, which has led to a high-level of refinement success (9,10).The most successful human refinement group in both CASP10 and CASP11 was the Feig group (1,11). Despite the relatively high performance of such strategies, using highly intensive MDS approaches for refinement has some drawbacks. The refinement procedure used by the Feig group consumed 75,000 core hours (12 days on 265 cores) on the multicore Intel Xenon CPU machines of the day, just to refine a single 3D model for a single protein target (12). Given that each protein structure prediction method will often produce hundreds or thousands of alternative models for each target, using similarly intensive MDS refinement procedures would quickly become problematic to manage in terms of CPU/GPU loads, if it were to be performed on multiple targets and models. This means that MDS refinement protocols, such as those developed by the Feig group, would be less practical to be used routinely for large scale fully automated structure prediction pipelines. Thus, faster, more practical and, ideally, as accurate and consistent, fully automated refinement servers are required. While many automated servers exists, few servers make use of the power of MDS and few adequately evaluate the models and present users with accurate reports of the likely improvement in errors. User feedback on when and, more specifically, where a 3D model has been improved is important to get right and it is often neglected.

We have a good track record in building 3D models (13–15) and estimating the likely errors they may have, recently termed Accuracy Self Estimates or (ASE) (16–18). We have had some success at using quality assessment guided multiple template based modelling to improve upon errors in single template models (19), but this approach requires sequence-structure alignments and template data. The ReFOLD server is our first successful attempt at developing a method for directly fixing likely errors in any user supplied 3D models, using global quality assessment guided refinement. The ReFOLD server also equips users with the ability to identify specific domains or regions in a protein that are likely to be correctly refined, via its accurate per-residue error estimates.

## MATERIALS AND METHODS

ReFOLD is the server implementation of the refinement method we initially developed for the CASP12 experiment. The ReFOLD method works using a unique hybrid approach consisting of rapid iterative refinement with i3Drefine (7) and molecular dynamics simulations with NAMD (20), combined with the latest version of our leading model quality estimation method, ModFOLD (3) (http://www.reading.ac.uk/bioinf/ModFOLD/, manuscript for version 6 in preparation). Input models were refined and evaluated over a number of successive stages. This iterative filtering process led to the generation of hundreds of alternative refined models, which were then ranked by quality.

The ReFOLD pipeline consisted of three protocols outlined in the flowchart shown in Supplementary Figure S1. The first protocol simply used a rapid iterative strategy for refinement of starting models, with 20 refinement cycles (iterations) of i3Drefine (7). Although the authors recommend not to run i3Drefine for more than 10 iterations, an optimal number of iterations is not specified, and often models will improve further beyond 10 iterations. Simply, all i3Drefine requires is a starting model, in PDB format, and a given number iterations as input parameters.

The second protocol employed a more complex and CPU/GPU intensive molecular dynamic simulation strategy, using NAMD (20) to refine each starting model. The NAMD protocol that we implemented was inspired by that of Feig and Mirjalili (10), utilizing all atom MD sampling in explicit solvent. Simulations were conducted at 298k under neutralized pH conditions with 1 bar of atmospheric pressure to resemble normal cellular conditions. Weak harmonic restraints with a spring constant of 0.05 kcal/mol/A^2^ on all C-alpha atoms were added to conserve aspects of the starting model. The CHARMM22/27 (21) force field (for protein systems, the two are equivalent) was used as the parameter file with default TIP3P water model (22). As some proteins are sensitive to ionic conditions in the solvent and PME (Particle Mesh Ewald) (23) was being used, the system was neutralized by adding either Na+ or Cl- ions. Only non-bonded interactions were calculated with bonded interactions excluded; the exclusion parameter was set to exclude up to four pairs of bonded atoms. Electrostatic and van der Waal interactions for these atom pairs were instead calculated using the parameter file (CHARMM27). A 12 Å cut-off for calculating non-bonded interactions (mostly van der Waal's) was applied, as this is the official standard for CHARMM force fields, with the smoothing function switching distance of 10 Å to avoid discontinuity in energy and forces. The *pairlistdist* function was set to a distance of 14 Å between atom pairs for inclusion in pair lists. All hydrogen bonding was rigidified, using the *rigidBonds* functions, allowing the time step to be increased to 2 fs. PME was used to calculate full electrostatics and the temperature in the system was controlled through Langevin dynamics (24), which balances random noise with friction to push atoms to the target temperature (298 K). The *langevinDamping* function was set to 1/ps to give some temperature control without dampening the system to the extent that effects were not seen. Periodic boundary conditions were used to achieve maintenance of conditions such as pressure, density and water box and also enable the use of PME, making the simulation more biologically realistic. The first stage of the MD simulation was 1000 steps of minimization, to lower the potential energy of the system reducing bad initial contacts, high force and temperature regions. This process zeros velocity, so that the *reinitvels* function returns the system to the desired temperature (298 K). For CASP12, the second protocol was run using multiple short trajectories in place of a single long trajectory; four parallel simulations were run for 2 ns, giving a cumulative simulation time of 8 ns per target.

The 164 refined models generated from the second protocol were assessed and ranked using ModFOLD6_rank method. The third protocol was a combination of the first two approaches, where the top ranked model from the second protocol was further refined using 20 iterations of i3Drefine. Subsequently, all of the refined models generated by each of these protocols and the starting model were pooled and re-ranked again using ModFOLD6_rank and the final top models were identified. Finally, each refined model was evaluated and compared with the original starting model in terms of local and global model quality scores. Static and dynamic graphical outputs were generated using the raw QA scores in order to display the top refined models and estimated improvements in a user friendly manner.

## RESULTS AND DISCUSSION

### Server inputs and outputs

**Figure 1.**
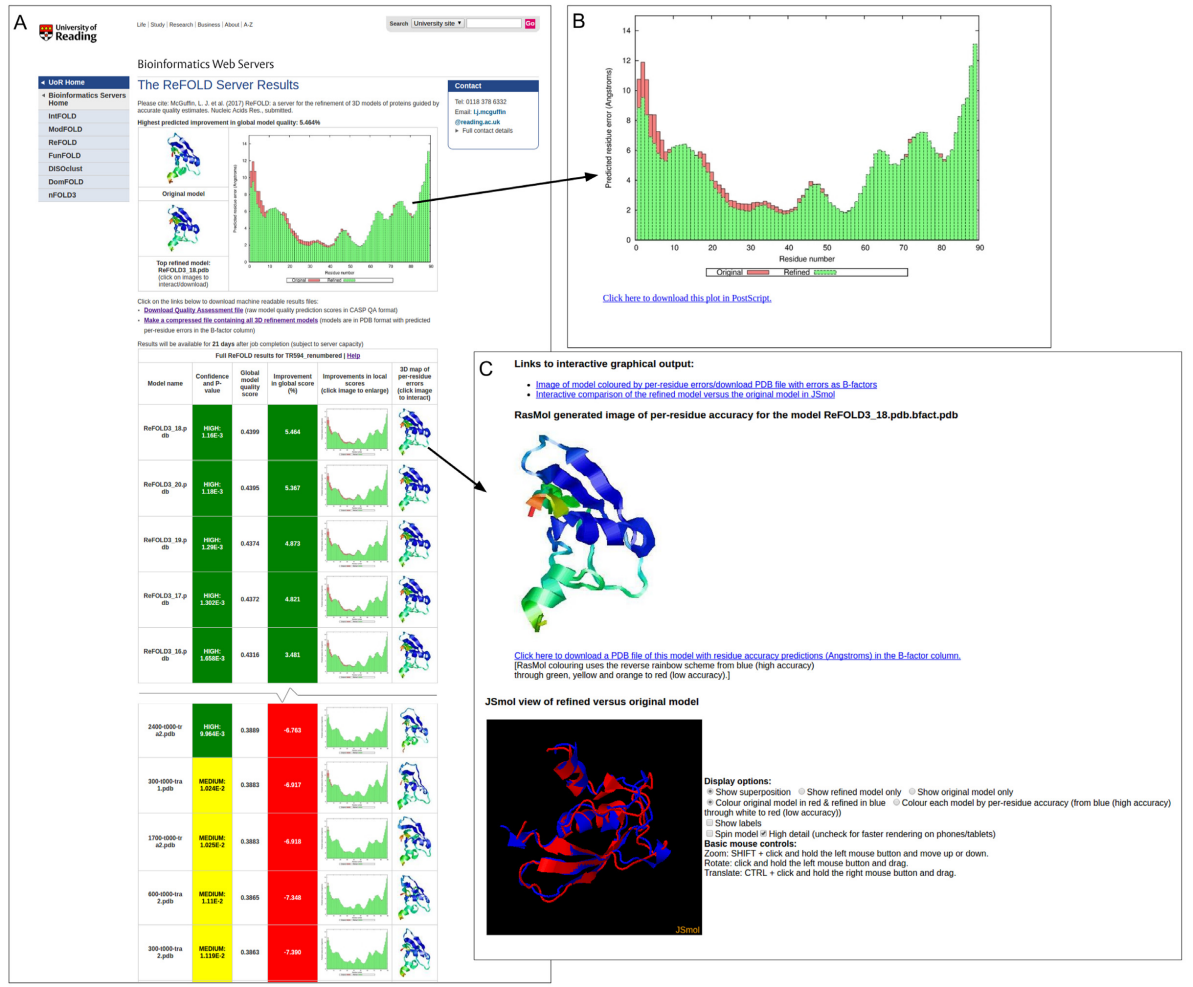
ReFOLD server results for CASP12 refinement target TR594. In CASP12, the McGuffin group used ReFOLD to improve the GDT_TS score from 55.34 → 58.43 (**A**) Main results page with a summary of the scores for the top refined model with the highest predicted improvement in model quality. The full table of scores for every alternative refined model is displayed below the top hit (truncated here to fit page). Clicking on the images on the main results page allows results to be visualized in more detail and downloaded. (**B**) Histogram of the local or per-residue ModFOLD6 errors for the top refined model (green bars) compared with the original model. Plots for each alternative refined model may be downloaded. (**C**) Interactive views of the refined model compared with the original model, which can be manipulated in 3D using the JSmol/HTML5 framework and/or downloaded for local viewing.

The only required inputs to the ReFOLD server are the amino acid sequence for the target protein and a single 3D model (in PDB format) for refinement. Users may optionally provide a name for their protein sequence and their email address. The ReFOLD server results page provides users with an accurate estimate of the likely percentage improvement in their global quality score based on the top refined model (Figure 1A). In addition, the server is unique in providing output for multiple alternative refined models in a way that allows users to quickly visualize the key residue locations, which are likely to have been improved upon compared to their original model. The results page provides users with a series of per-residue error plots, which demonstrate the reduction in local errors in the refined models compared with the uploaded original (Figure 1B). This is important, as global refinement of a full chain model may not always occur, whereas local regions, or individual domains, may often be much improved. Presenting results to users in this way also gives them the choice to easily compare alternative refined models, allowing them to focus their attention to key interacting residues or specific domains. Users can also click through the images in the table in order to compare the refined and original 3D models interactively in using the JSmol/HTML5 framework (Figure 1C). No plugins are required and, conveniently, interactive results may also be viewed on mobile devices.

### Independent benchmarking

Our ReFOLD refinement approach was independently tested by the assessor team in the recent CASP12 experiment, and it was a key factor contributing to our success. During the CASP12 prediction season (May–August 2016), we used ReFOLD to build hundreds of alternative refined models, for both the main tertiary structure prediction and refinement categories. ReFOLD gave us a significant performance boost in the main tertiary structure prediction category, where it enabled us to further improve the quality of some of the very best initial server models. As a result of our high performance, we were invited to speak at the meeting in Gaeta about our template based modelling (TBM) strategy. Our group ranked in 2nd position overall on both the TBM and TBM/FM (Free Modelling) targets according to the assessors’ formula (Supplementary Tables S1 and S2), and we ranked 11th overall on FM targets. Our group also improved the global and local quality scores for many of the starting models provided in the refinement category itself, where we ranked 14th overall (http://predictioncenter.org/casp12/).

**Figure 2.**
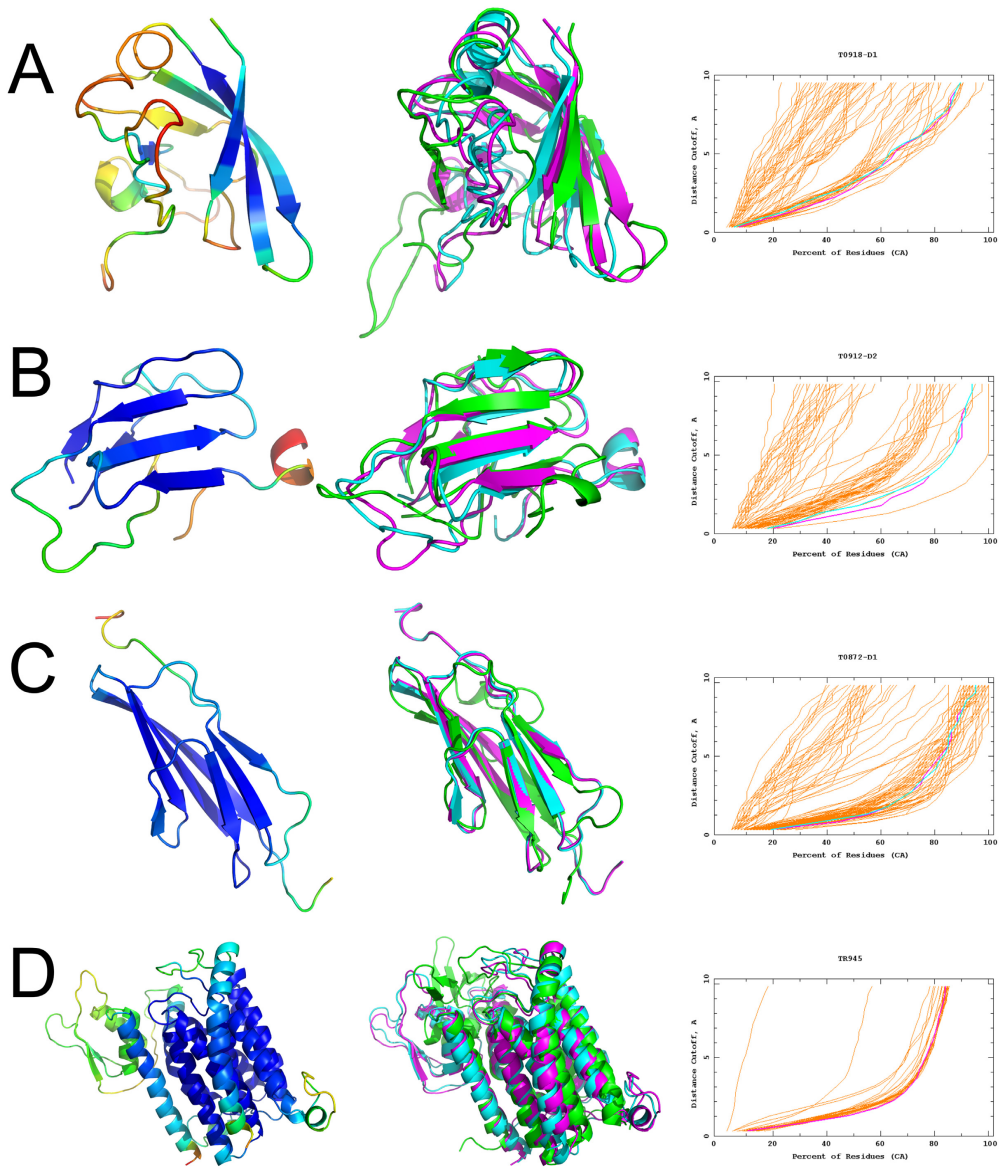
Four examples of CASP12 targets where using ReFOLD allowed us to fix errors in the top selected server model. Left panels, refined model with ModFOLD6 accuracy self assessment (ASE) displayed using the temperature colour scheme. Middle panels, superposition of the top selected server model (cyan), refined model (magenta) and native structure (green). Right panels, GDT plots comparing top selected server models (cyan) with the ReFOLD refined models (magenta). (**A**) FM target T0918 domain 1: QUARK_TS1 versus McGuffin_TS1, a GDT_TS improvement from 45.6 to 48.38. (**B**) FM/TBM target T0912 domain 2: GOAL_TS1 versus McGuffin_TS1, GDT_TS from 62.95 to 65.36. (**C**) TBM target T0872 domain 1: IntFOLD4_TS1 versus McGuffin_TS1, GDT_TS from 66.76 to 67.9 (**D**) TR945: starting model versus McGuffin_TS1, GDT_TS from 59.27 to 61.20. Models are rendered using PyMOL (http://www.pymol.org). GDT plots are from http://www.predictioncenter.org/casp12/.

The results in Table 1 summarize our CASP12 results for regular targets, where the top server models, selected by ModFOLD_rank, were then further refined with ReFOLD. Arguably, this benchmark represents a more realistic user test case, where each of the starting models have been selected in a fully automated manner and have been generated for full length protein chains. The results in Table 1 show that the majority of models were either improved upon or unchanged according to the GDT_TS (25) and MolProbity (26) scores. There is an average overall improvement in scores (∑ΔGDT_TS = 8.15, ∑ΔMolProbity = –10.16), which are greater than the standard errors, and there is a strong correlation between the ModFOLD6_rank global scores and GDT_TS scores (Pearson's *r* = 0.8094). The GDT_TS score measures the global positioning of the backbone C-alpha atoms based on multiple superpositions of the predicted and experimental structure. It is promising to see that ReFOLD improves backbone quality, but these changes are relatively small, so significant improvements in GDT_TS are hard to detect on the CASP data set. While it is encouraging that the improvement in cumulative GDT_TS scores is greater than the error, the pairwise t-test on the data in Table 1 does not provide evidence that the improvement in the backbone is statistically significant (*P* = 0.1196). However, the improvement in the MolProbity full atom scores is statistically significant at the 95% confidence level, according to the pairwise t-test on the data in Table 1 (*P* = 0.0301). Whereas the GDT_TS score focuses only on the C-alpha atoms, the MolProbity score is an all-atom composite score, which means that it also measures the finer detail of differences in the local errors of the side chains. The MolProbity score denotes the expected resolution with respect to experimental structures, therefore models with *lower* MolProbity scores are more physically realistic. Figure 2 shows examples where ReFOLD has successfully refined starting models of varying target difficulty (FM, TBM, TBM/FM targets and a refinement target), with improved GDT_TS scores for each.

**Table 1.** Summary of ReFOLD performance on CASP12 targets for full chains

	CASP model ID		ModFOLD6_rank		GDT-TS			MolProbity		
Target ID	Starting model	Refined model	Starting model	Refined model	Starting model	Refined model	Diff.	Starting model	Refined model	Diff.
T0859	T0859TS479_1-D1	T0859TS017_1-D1	0.4059	0.4189	24.78	27.66	2.88	2.88	2.7	–0.18
T0862	T0862TS183_3-D1	T0862TS017_1-D1	0.3809	0.3809	58.6	58.6	0	2.66	2.66	0
T0863	T0863TS005_1	T0863TS017_1	0.2994	0.3024	9.62	9.54	–0.08	0.93	1.61	0.68
T0864	T0864TS479_4-D1	T0864TS017_1-D1	0.3514	0.3658	20.22	19.92	–0.3	3.17	1.85	–1.32
T0866	T0866TS479_5-D1	T0866TS017_1-D1	0.4049	0.425	44.71	45.91	1.2	3.44	2.45	–0.99
T0868	T0868TS479_3-D1	T0868TS017_1-D1	0.4338	0.4438	57.97	60.99	3.02	2.83	0.65	–2.18
T0869	T0869TS479_3-D1	T0869TS017_1-D1	0.4288	0.432	32.21	31.97	–0.24	3.17	3.79	0.62
T0870	T0870TS183_4-D1	T0870TS017_1-D1	0.4733	0.4804	33.94	32.32	–1.62	2.86	1.07	–1.79
T0872	T0872TS405_1-D1	T0872TS017_1-D1	0.6467	0.6539	66.76	67.9	1.14	2.63	3.12	0.49
T0874	T0874TS005_2-D1	T0874TS017_1-D1	0.512	0.5148	46.17	46.17	0	0.92	2.1	1.18
T0875	T0875TS005_2-D1	T0875TS017_1-D1	0.5254	0.53	43.1	43.1	0	1.19	1.19	0
T0876	T0876TS220_1-D1	T0876TS017_1-D1	0.4563	0.4684	48.54	49.38	0.84	1.9	2.41	0.51
T0878	T0878TS220_2-D1	T0878TS017_1-D1	0.3544	0.364	13.01	12.5	–0.51	2.66	2.09	–0.57
T0880	T0880TS405_1	T0880TS017_1	0.3885	0.4201	10.75	11.27	0.52	3.07	2.15	–0.92
T0882	T0882TS357_4-D1	T0882TS017_1-D1	0.6089	0.6161	79.43	81.33	1.9	1.73	1.36	–0.37
T0886	T0886TS479_1	T0886TS017_1	0.3569	0.3758	26.96	28.27	1.31	3.19	2.91	–0.28
T0887	T0887TS220_2-D1	T0887TS017_1-D1	0.4649	0.4756	40.22	38.51	–1.71	1.79	1.44	–0.35
T0888	T0888TS183_4-D1	T0888TS017_1-D1	0.3677	0.3778	19.84	19.21	–0.63	3	3.93	0.93
T0890	T0890TS220_2	T0890TS017_1	0.458	0.4653	25.53	24.73	–0.8	2.27	1.17	–1.1
T0892	T0892TS479_3	T0892TS017_1	0.4193	0.4295	37.18	38.34	1.16	3.03	2.01	–1.02
T0894	T0894TS183_1	T0894TS017_1	0.3434	0.3469	50.52	51.05	0.53	3.57	3.81	0.24
T0895	T0895TS250_5-D1	T0895TS017_1-D1	0.584	0.584	71.67	71.67	0	2.32	2.32	0
T0896	T0896TS220_2	T0896TS017_1	0.3193	0.3252	19.63	20.75	1.12	1.94	1.93	–0.01
T0897	T0897TS005_2	T0897TS017_1	0.3141	0.3179	10.02	9.64	–0.38	1.03	1.79	0.76
T0898	T0898TS405_1	T0898TS017_1	0.39	0.4062	28.42	28.11	-0.31	3.47	3.63	0.16
T0899	T0899TS183_2	T0899TS017_1	0.3994	0.4062	27.88	28.1	0.22	3.69	3.02	–0.67
T0900	T0900TS479_1-D1	T0900TS017_1-D1	0.4995	0.5129	42.65	44.85	2.2	3.13	1.05	–2.08
T0901	T0901TS479_2	T0901TS017_1	0.4908	0.4952	37.37	37.2	–0.17	3.37	3.82	0.45
T0904	T0904TS479_1-D1	T0904TS017_1-D1	0.4628	0.4656	38.74	38.55	–0.19	3.2	2.63	–0.57
T0905	T0905TS479_2	T0905TS017_1	0.5289	0.531	35.06	34.98	–0.08	3.23	2.87	–0.36
T0907	T0907TS479_1	T0907TS017_1	0.3736	0.391	21.32	20.55	–0.77	3.41	2.82	–0.59
T0909	T0909TS251_5-D1	T0909TS017_1-D1	0.44	0.4485	41.07	41.07	0	3.32	2.59	–0.73
T0911	T0911TS405_2-D1	T0911TS017_1-D1	0.6009	0.6053	52.02	52.63	0.61	3.37	3.4	0.03
T0912	T0912TS220_1	T0912TS017_1	0.4187	0.4237	47.66	47.16	-0.5	2.36	1.87	–0.49
T0913	T0913TS479_5-D1	T0913TS017_1-D1	0.5734	0.5762	61.98	62.06	0.08	3.06	3.75	0.69
T0914	T0914TS183_1	T0914TS017_1	0.349	0.349	16.33	16.33	0	3.71	3.71	0
T0915	T0915TS220_1-D1	T0915TS017_1-D1	0.4873	0.4988	48.86	47.4	–1.46	1.85	1.94	0.09
T0918	T0918TS183_1	T0918TS017_1	0.3226	0.3315	14.83	15.54	0.71	3.25	2.11	–1.14
T0923	T0923TS220_2-D1	T0923TS017_1-D1	0.3305	0.3334	18.18	18.18	0	1.92	1.24	–0.68
T0941	T0941TS183_5-D1	T0941TS017_1-D1	0.3366	0.3465	8.94	9.16	0.22	3.01	3.73	0.72
T0942	T0942TS183_1	T0942TS017_1	0.4443	0.4497	45.54	45.22	-0.32	2.78	3.4	0.62
T0944	T0944TS220_5-D1	T0944TS017_1-D1	0.6205	0.6246	72.92	73.22	0.3	1.87	1.67	–0.2
T0945	T0945TS183_1-D1	T0945TS017_1-D1	0.4438	0.4536	53.8	53.8	0	3.73	3.73	0
T0946	T0946TS479_1	T0946TS017_1	0.4508	0.4572	45.12	44.86	–0.26	3.73	3.84	0.11
T0947	T0947TS220_1-D1	T0947TS017_1-D1	0.5149	0.5277	64.43	63.29	–1.14	2.01	2.76	0.75
T0948	T0948TS479_1-D1	T0948TS017_1-D1	0.6367	0.6368	71.98	71.64	–0.34	2.69	2.09	–0.6
				Total	1786.48	1794.63	8.15	124.34	114.18	–10.16
				Std. Err.	2.822	2.845	–	0.1152	0.1367	–

Mean GDT_TS of starting model = 38.84. ∑ΔGDT_TS = 8.15 (higher scores are better). Mean MolProbity of starting model = 2.70. ∑ΔMolProbity = –10.16 (lower scores are better). Data are from http://www.predictioncenter.org/casp12/.

It is clear that the success of refinement is related to the quality of the starting model, when targets are subdivided into domains (Supplementary Tables S3–S5). Dividing targets into domains allows us to pinpoint where the method performance is strongest. The results indicate that the method works better overall on harder FM targets, which have on average lower initial GDT_TS scores. The method is less successful on the assessor selected and edited refinement targets and the TBM domain models (where starting models have, on average, much higher GDT_TS scores) than either the full chain models or FM domain models. Clearly, it is harder to refine models where there is less room for improvement, and likewise it is harder to detect a smaller ΔGDT_TS. In addition, for many of the starting server models, the developers also attempted refinement, which clearly produces a problem of diminishing returns for further refinement. Nevertheless, considering full chain models across regular targets, on average the automatically selected initial models are successfully improved upon by the ReFOLD pipeline.

The time taken to refine a model is dependent on the sequence length. The average time per CASP12 target was ∼10 h for each NAMD run using a quad core desktop (Intel Xeon E5-2407v2) with a standard graphics card (Nvidia GeForce GTX 970). Smaller models were quicker to refine (e.g. 310 min for a 60 residue model) and larger models took longer (e.g. 909 min for a 257 residue model). The other components of the method, including the quality assessment, are run in parallel and will usually take no more than a few extra hours. The ReFOLD pipeline is presently running on a dedicated 56 thread server (Intel Xeon E5-2695 v3) with an Nvidia Tesla K40M GPU card, so most users should expect their results well within 24 h, once they are running.

## CONCLUSIONS

The ReFOLD server allows users to attempt to fix errors in their 3D models of proteins and identify improvements with built-in Accuracy Self Estimates. The user friendly, dynamic results pages let users visualise potential improvements for over 200 alternative refined models, at both a global and local level. Providing users with visual comparisons of estimated local improvement allows them to quickly identify those models, which are likely to have been improved upon in a specific region of interest. In addition, the server provides users with a compressed archive all of the generated refined models, which they may rank using their own alternative quality assessment protocols. In the recent CASP12 experiment, the ReFOLD pipeline gave our group a performance boost increasing our cumulative GDT_TS scores and thereby contributing to our high overall rankings.

## Supplementary Material

Supplementary Data
