# Supplementary material for: ReFOLD: a server for the refinement of 3D protein models guided by accurate quality estimates
Source: Nucleic Acids Res. 2017 Apr 10;45(Web Server issue):W422–8. doi: 10.1093/nar/gkx249 (PMC5570150; doi:10.1093/nar/gkx249)
Supplement: Supplementary Data [file gkx249_Supp.docx]

Supplementary Figure S1. Flow of data and methods used in the ReFOLD server. The starting model is initially processed using a conservative fast iterative protocol (i3Drefine) and a slower more speculative molecular dynamics simulation protocol (NAMD). Models from the NAMD protocol are ranked using ModFOLD6_rank and the top model is selected for a further round of refinement using i3Drefine. Finally all refined models are pooled and ranked using ModFOLD6_rank, the scores are then compared with the original starting model and the results are displayed to the user.


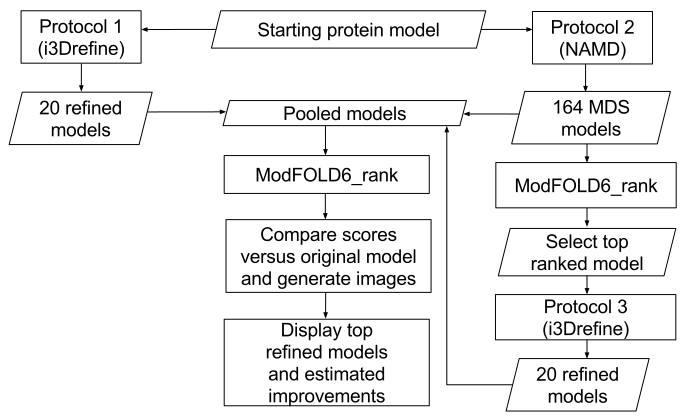


Supplementary Table S1. Official CASP12 results summary for TBM + TBM/FM domains using the Assessors’ formula (GDT_HA + ASE). The top 20 groups are shown. Data are from <http://www.predictioncenter.org/casp12/>.

| Rank | GR  code | GR  name | Domains Count | SUM Zscore  (>-2.0) | Rank SUM Zscore  (>-2.0) | AVG Zscore  (>-2.0) | Rank AVG Zscore  (>-2.0) | SUM Zscore  (>0.0) | Rank SUM Zscore  (>0.0) | AVG Zscore  (>0.0) | Rank AVG Zscore  (>0.0) |
| --- | --- | --- | --- | --- | --- | --- | --- | --- | --- | --- | --- |
| 1 | 4 | Zhang | 29 | 27.7458 | 1 | 0.9568 | 1 | 28.0675 | 1 | 0.9678 | 1 |
| **2** | **17** | **McGuffin** | **29** | **26.3653** | **2** | **0.9091** | **2** | **27.3813** | **2** | **0.9442** | **2** |
| 3 | 417 | VoroMQA-select | 29 | 25.0555 | 3 | 0.864 | 3 | 25.8104 | 3 | 0.89 | 3 |
| 4 | 479 | Zhang-Server | 29 | 24.276 | 4 | 0.8371 | 4 | 24.6911 | 4 | 0.8514 | 5 |
| 5 | 183 | QUARK | 29 | 22.4209 | 5 | 0.7731 | 5 | 23.0321 | 8 | 0.7942 | 9 |
| 6 | 73 | Wallner | 29 | 22.3665 | 6 | 0.7713 | 6 | 23.1761 | 7 | 0.7992 | 8 |
| 7 | 439 | MULTICOM | 29 | 21.3858 | 7 | 0.7374 | 8 | 22.0663 | 9 | 0.7609 | 10 |
| 8 | 203 | ProQ2 | 29 | 20.9704 | 8 | 0.7231 | 9 | 23.4112 | 6 | 0.8073 | 7 |
| 9 | 252 | wfRosetta-ProQ-ModF6 | 28 | 19.382 | 9 | 0.7636 | 7 | 23.9838 | 5 | 0.8566 | 4 |
| 10 | 405 | IntFOLD4 | 29 | 18.3286 | 10 | 0.632 | 10 | 19.607 | 11 | 0.6761 | 14 |
| 11 | 243 | Seok-refine | 29 | 16.9753 | 11 | 0.5854 | 13 | 20.8213 | 10 | 0.718 | 12 |
| 12 | 64 | Jones-UCL | 29 | 13.5821 | 12 | 0.4683 | 16 | 17.1937 | 17 | 0.5929 | 20 |
| 13 | 250 | Seok-server | 29 | 13.3768 | 13 | 0.4613 | 17 | 17.5751 | 15 | 0.606 | 19 |
| 14 | 23 | Seok | 28 | 13.2878 | 14 | 0.546 | 14 | 17.4489 | 16 | 0.6232 | 18 |
| 15 | 236 | MULTICOM-CONSTRUCT | 29 | 13.0953 | 15 | 0.4516 | 18 | 14.843 | 24 | 0.5118 | 28 |
| 16 | 320 | raghavagps | 28 | 12.4814 | 16 | 0.5172 | 15 | 18.9179 | 12 | 0.6756 | 15 |
| 17 | 239 | wfAll-Cheng | 27 | 12.2087 | 17 | 0.6003 | 11 | 18.1602 | 14 | 0.6726 | 16 |
| 18 | 345 | MULTICOM-NOVEL | 29 | 11.4981 | 18 | 0.3965 | 21 | 13.1076 | 29 | 0.452 | 38 |
| 19 | 287 | MULTICOM-CLUSTER | 29 | 11.4089 | 19 | 0.3934 | 22 | 14.1322 | 26 | 0.4873 | 31 |
| 20 | 60 | KIAS-Gdansk | 29 | 8.2106 | 20 | 0.2831 | 25 | 16.5924 | 18 | 0.5722 | 22 |

Supplementary Table S2 Official CASP12 results summary for TBM domains using the Assessors’ formula (GDT_HA + ASE). The top 20 groups are shown. Data are from <http://www.predictioncenter.org/casp12/>.

| Rank | GR  code | GR  name | Domains Count | SUM Zscore  (>-2.0) | Rank SUM Zscore  (>-2.0) | AVG Zscore  (>-2.0) | Rank AVG Zscore  (>-2.0) | SUM Zscore  (>0.0) | Rank SUM Zscore  (>0.0) | AVG Zscore  (>0.0) | Rank AVG Zscore  (>0.0) |
| --- | --- | --- | --- | --- | --- | --- | --- | --- | --- | --- | --- |
| 1 | 4 | Zhang | 12 | 11.9816 | 1 | 0.9985 | 1 | 11.9816 | 1 | 0.9985 | 1 |
| **2** | **17** | **McGuffin** | **12** | **11.3106** | **2** | **0.9425** | **2** | **11.4266** | **3** | **0.9522** | **3** |
| 3 | 252 | wfRosetta-ProQ-ModF6 | 12 | 11.155 | 3 | 0.9296 | 3 | 11.5591 | 2 | 0.9633 | 2 |
| 4 | 203 | ProQ2 | 12 | 11.153 | 4 | 0.9294 | 4 | 11.153 | 4 | 0.9294 | 4 |
| 5 | 417 | VoroMQA-select | 12 | 10.9344 | 5 | 0.9112 | 5 | 10.9344 | 5 | 0.9112 | 5 |
| 6 | 479 | Zhang-Server | 12 | 10.8281 | 6 | 0.9023 | 6 | 10.8281 | 6 | 0.9023 | 6 |
| 7 | 183 | QUARK | 12 | 10.2454 | 7 | 0.8538 | 7 | 10.2454 | 7 | 0.8538 | 7 |
| 8 | 73 | Wallner | 12 | 10.1708 | 8 | 0.8476 | 8 | 10.1708 | 8 | 0.8476 | 8 |
| 9 | 243 | Seok-refine | 12 | 9.5708 | 9 | 0.7976 | 9 | 9.5708 | 10 | 0.7976 | 10 |
| 10 | 64 | Jones-UCL | 12 | 9.3655 | 10 | 0.7805 | 10 | 9.8517 | 9 | 0.821 | 9 |
| 11 | 439 | MULTICOM | 12 | 8.7888 | 11 | 0.7324 | 11 | 8.7888 | 11 | 0.7324 | 13 |
| 12 | 405 | IntFOLD4 | 12 | 8.5739 | 12 | 0.7145 | 13 | 8.7324 | 12 | 0.7277 | 14 |
| 13 | 320 | raghavagps | 12 | 7.3498 | 13 | 0.6125 | 16 | 8.6225 | 13 | 0.7185 | 16 |
| 14 | 287 | MULTICOM-CLUSTER | 12 | 6.9947 | 14 | 0.5829 | 17 | 7.0564 | 21 | 0.588 | 25 |
| 15 | 60 | KIAS-Gdansk | 12 | 6.6919 | 15 | 0.5577 | 19 | 7.3253 | 20 | 0.6104 | 23 |
| 16 | 236 | MULTICOM-CONSTRUCT | 12 | 6.493 | 16 | 0.5411 | 20 | 6.8271 | 22 | 0.5689 | 27 |
| 17 | 250 | Seok-server | 12 | 6.1965 | 17 | 0.5164 | 21 | 7.7518 | 19 | 0.646 | 21 |
| 18 | 23 | Seok | 11 | 5.9453 | 18 | 0.7223 | 12 | 8.4206 | 15 | 0.7655 | 12 |
| 19 | 239 | wfAll-Cheng | 11 | 5.3797 | 19 | 0.6709 | 14 | 7.9237 | 16 | 0.7203 | 15 |
| 20 | 345 | MULTICOM-NOVEL | 12 | 5.1329 | 20 | 0.4277 | 24 | 5.579 | 31 | 0.4649 | 42 |

Supplementary Table S3. Summary of ReFOLD performance on CASP12 FM domains. Mean GDT_TS of starting model = 31.3875. ∑∆GDT_TS = 3.3. Mean MolProbity score of starting model = 2.7965. ∑∆MolProbity = -11.74. Data are from <http://www.predictioncenter.org/casp12/>.

|  | CASP model ID | | GDT-TS | | | MolProbity | | |
| --- | --- | --- | --- | --- | --- | --- | --- | --- |
| Target ID by domain | Starting model | Refined model | Starting model | Refined model | Diff. | Starting model | Refined model | Diff. |
| T0859-D1 | T0859TS479_1-D1 | T0859TS017_1-D1 | 24.78 | 27.66 | 2.88 | 2.88 | 2.7 | -0.18 |
| T0862-D1 | T0862TS183_3-D1 | T0862TS017_1-D1 | 58.6 | 58.6 | 0 | 2.66 | 2.66 | 0 |
| T0863-D1 | T0863TS005_1-D1 | T0863TS017_1-D1 | 16.84 | 16.45 | -0.39 | 0.74 | 1.37 | 0.63 |
| T0863-D2 | T0863TS005_1-D2 | T0863TS017_1-D2 | 14.96 | 14.54 | -0.42 | 0.87 | 1.67 | 0.8 |
| T0864-D1 | T0864TS479_4-D1 | T0864TS017_1-D1 | 20.22 | 19.92 | -0.3 | 3.17 | 1.85 | -1.32 |
| T0866-D1 | T0866TS479_5-D1 | T0866TS017_1-D1 | 44.71 | 45.91 | 1.2 | 3.44 | 2.45 | -0.99 |
| T0869-D1 | T0869TS479_3-D1 | T0869TS017_1-D1 | 32.21 | 31.97 | -0.24 | 3.17 | 3.79 | 0.62 |
| T0870-D1 | T0870TS183_4-D1 | T0870TS017_1-D1 | 33.94 | 32.32 | -1.62 | 2.86 | 1.07 | -1.79 |
| T0878-D1 | T0878TS220_2-D1 | T0878TS017_1-D1 | 13.01 | 12.5 | -0.51 | 2.66 | 2.09 | -0.57 |
| T0880-D1 | T0880TS405_1-D1 | T0880TS017_1-D1 | 31.25 | 29.86 | -1.39 | 3.11 | 2.11 | -1 |
| T0880-D2 | T0880TS405_1-D2 | T0880TS017_1-D2 | 13.22 | 13.22 | 0 | 3.09 | 2.1 | -0.99 |
| T0886-D1 | T0886TS479_1-D1 | T0886TS017_1-D1 | 41.67 | 41.67 | 0 | 2.9 | 2.87 | -0.03 |
| T0886-D2 | T0886TS479_1-D2 | T0886TS017_1-D2 | 48.62 | 50.79 | 2.17 | 3.3 | 2.98 | -0.32 |
| T0888-D1 | T0888TS183_4-D1 | T0888TS017_1-D1 | 19.84 | 19.21 | -0.63 | 3 | 3.93 | 0.93 |
| T0890-D2 | T0890TS220_2-D2 | T0890TS017_1-D2 | 30.9 | 30.19 | -0.71 | 2.35 | 1.55 | -0.8 |
| T0892-D2 | T0892TS479_3-D2 | T0892TS017_1-D2 | 40.91 | 40.23 | -0.68 | 3.17 | 2.05 | -1.12 |
| T0894-D1 | T0894TS183_1-D1 | T0894TS017_1-D1 | 57.02 | 58.71 | 1.69 | 2.77 | 3.45 | 0.68 |
| T0896-D3 | T0896TS220_2-D3 | T0896TS017_1-D3 | 12.27 | 12.11 | -0.16 | 1.68 | 1.9 | 0.22 |
| T0897-D1 | T0897TS005_2-D1 | T0897TS017_1-D1 | 16.3 | 16.85 | 0.55 | 0.8 | 1.62 | 0.82 |
| T0897-D2 | T0897TS005_2-D2 | T0897TS017_1-D2 | 18.75 | 18.75 | 0 | 1.09 | 1.76 | 0.67 |
| T0898-D1 | T0898TS405_1-D1 | T0898TS017_1-D1 | 34.91 | 34.2 | -0.71 | 3.48 | 3.59 | 0.11 |
| T0899-D1 | T0899TS183_2-D1 | T0899TS017_1-D1 | 36.58 | 36.78 | 0.2 | 3.82 | 3.13 | -0.69 |
| T0899-D2 | T0899TS183_2-D2 | T0899TS017_1-D2 | 24.72 | 24.72 | 0 | 3.39 | 2.81 | -0.58 |
| T0900-D1 | T0900TS479_1-D1 | T0900TS017_1-D1 | 42.65 | 44.85 | 2.2 | 3.13 | 1.05 | -2.08 |
| T0901-D2 | T0901TS479_2-D2 | T0901TS017_1-D2 | 24.29 | 25 | 0.71 | 3.07 | 3.75 | 0.68 |
| T0904-D1 | T0904TS479_1-D1 | T0904TS017_1-D1 | 38.74 | 38.55 | -0.19 | 3.2 | 2.63 | -0.57 |
| T0905-D1 | T0905TS479_2-D1 | T0905TS017_1-D1 | 42.25 | 42.35 | 0.1 | 3.06 | 2.77 | -0.29 |
| T0905-D2 | T0905TS479_2-D2 | T0905TS017_1-D2 | 24.62 | 24.62 | 0 | 2.93 | 2.29 | -0.64 |
| T0907-D3 | T0907TS479_1-D3 | T0907TS017_1-D3 | 40.17 | 40.38 | 0.21 | 3.02 | 2.51 | -0.51 |
| T0912-D3 | T0912TS220_1-D3 | T0912TS017_1-D3 | 21.84 | 20.63 | -1.21 | 2.38 | 1.41 | -0.97 |
| T0914-D1 | T0914TS183_1-D1 | T0914TS017_1-D1 | 31.01 | 31.01 | 0 | 3.56 | 3.56 | 0 |
| T0914-D2 | T0914TS183_1-D2 | T0914TS017_1-D2 | 26.85 | 26.85 | 0 | 3.22 | 3.22 | 0 |
| T0915-D1 | T0915TS220_1-D1 | T0915TS017_1-D1 | 48.86 | 47.4 | -1.46 | 1.85 | 1.94 | 0.09 |
| T0918-D1 | T0918TS183_1-D1 | T0918TS017_1-D1 | 45.6 | 48.38 | 2.78 | 3.68 | 2.32 | -1.36 |
| T0918-D2 | T0918TS183_1-D2 | T0918TS017_1-D2 | 34.35 | 34.96 | 0.61 | 3.12 | 2.19 | -0.93 |
| T0918-D3 | T0918TS183_1-D3 | T0918TS017_1-D3 | 39.62 | 37.71 | -1.91 | 3.26 | 2.21 | -1.05 |
| T0923-D1 | T0923TS220_2-D1 | T0923TS017_1-D1 | 18.18 | 18.18 | 0 | 1.92 | 1.24 | -0.68 |
| T0941-D1 | T0941TS183_5-D1 | T0941TS017_1-D1 | 8.94 | 9.16 | 0.22 | 3.01 | 3.73 | 0.72 |
| T0945-D1 | T0945TS183_1-D1 | T0945TS017_1-D1 | 53.8 | 53.8 | 0 | 3.73 | 3.73 | 0 |
| T0946-D1 | T0946TS479_1-D1 | T0946TS017_1-D1 | 27.5 | 27.81 | 0.31 | 3.32 | 4.07 | 0.75 |

Supplementary Table S4. Summary of ReFOLD performance on CASP12 TBM domains. Mean GDT_TS of starting model = 66.61. ∑∆GDT_TS = 0.9. Mean MolProbity score of starting model = 2.5425. ∑∆MolProbity = 2.21. Data are from <http://www.predictioncenter.org/casp12/>.

|  | CASP model ID | | GDT-TS | | | MolProbity | | |
| --- | --- | --- | --- | --- | --- | --- | --- | --- |
| Target ID by domain | Starting model | Refined model | Starting model | Refined model | Diff. | Starting model | Refined model | Diff. |
| T0872-D1 | T0872TS405_1-D1 | T0872TS017_1-D1 | 66.76 | 67.9 | 1.14 | 2.63 | 3.12 | 0.49 |
| T0882-D1 | T0882TS357_4-D1 | T0882TS017_1-D1 | 79.43 | 81.33 | 1.9 | 1.73 | 1.36 | -0.37 |
| T0895-D1 | T0895TS250_5-D1 | T0895TS017_1-D1 | 71.67 | 71.67 | 0 | 2.32 | 2.32 | 0 |
| T0911-D1 | T0911TS405_2-D1 | T0911TS017_1-D1 | 52.02 | 52.63 | 0.61 | 3.37 | 3.4 | 0.03 |
| T0912-D1 | T0912TS220_1-D1 | T0912TS017_1-D1 | 61.05 | 60.45 | -0.6 | 2.22 | 1.87 | -0.35 |
| T0913-D1 | T0913TS479_5-D1 | T0913TS017_1-D1 | 61.98 | 62.06 | 0.08 | 3.06 | 3.75 | 0.69 |
| T0942-D1 | T0942TS183_1-D1 | T0942TS017_1-D1 | 79.91 | 79.33 | -0.58 | 2.24 | 3.34 | 1.1 |
| T0942-D2 | T0942TS183_1-D2 | T0942TS017_1-D2 | 55.49 | 55.49 | 0 | 2.96 | 3.4 | 0.44 |
| T0944-D1 | T0944TS220_5-D1 | T0944TS017_1-D1 | 72.92 | 73.22 | 0.3 | 1.87 | 1.67 | -0.2 |
| T0946-D2 | T0946TS479_1-D2 | T0946TS017_1-D2 | 61.67 | 61.2 | -0.47 | 3.41 | 3.64 | 0.23 |
| T0947-D1 | T0947TS220_1-D1 | T0947TS017_1-D1 | 64.43 | 63.29 | -1.14 | 2.01 | 2.76 | 0.75 |
| T0948-D1 | T0948TS479_1-D1 | T0948TS017_1-D1 | 71.98 | 71.64 | -0.34 | 2.69 | 2.09 | -0.6 |

Supplementary Table S5. Summary of ReFOLD performance on CASP12 FM/TBM domains. Mean GDT_TS of starting model = 55.69. ∑∆GDT_TS=-7.86. Mean MolProbity score of starting model = 2.3581. ∑∆MolProbity = -3.88. Data are from <http://www.predictioncenter.org/casp12/>.

|  | CASP model ID | | GDT-TS | | | MolProbity | | |
| --- | --- | --- | --- | --- | --- | --- | --- | --- |
| Target ID by domain | Starting model | Refined model | Starting model | Refined model | Diff. | Starting model | Refined model | Diff. |
| T0868-D1 | T0868TS479_3-D1 | T0868TS017_1-D1 | 57.97 | 60.99 | 3.02 | 2.83 | 0.65 | -2.18 |
| T0874-D1 | T0874TS005_2-D1 | T0874TS017_1-D1 | 46.17 | 46.17 | 0 | 0.92 | 2.1 | 1.18 |
| T0875-D1 | T0875TS005_2-D1 | T0875TS017_1-D1 | 43.1 | 43.1 | 0 | 1.19 | 1.19 | 0 |
| T0876-D1 | T0876TS220_1-D1 | T0876TS017_1-D1 | 48.54 | 49.38 | 0.84 | 1.9 | 2.41 | 0.51 |
| T0887-D1 | T0887TS220_2-D1 | T0887TS017_1-D1 | 40.22 | 38.51 | -1.71 | 1.79 | 1.44 | -0.35 |
| T0890-D1 | T0890TS220_2-D1 | T0890TS017_1-D1 | 58.23 | 55.79 | -2.44 | 1.78 | 0.5 | -1.28 |
| T0892-D1 | T0892TS479_3-D1 | T0892TS017_1-D1 | 79.71 | 82.25 | 2.54 | 2.22 | 1.72 | -0.5 |
| T0894-D2 | T0894TS183_1-D2 | T0894TS017_1-D2 | 73.15 | 72.69 | -0.46 | 2.67 | 2.96 | 0.29 |
| T0896-D1 | T0896TS220_2-D1 | T0896TS017_1-D1 | 54.94 | 50.58 | -4.36 | 2.38 | 1.54 | -0.84 |
| T0896-D2 | T0896TS220_2-D2 | T0896TS017_1-D2 | 42.12 | 45.25 | 3.13 | 1.72 | 1.98 | 0.26 |
| T0898-D2 | T0898TS405_1-D2 | T0898TS017_1-D2 | 49.09 | 49.55 | 0.46 | 3.43 | 3.67 | 0.24 |
| T0901-D1 | T0901TS479_2-D1 | T0901TS017_1-D1 | 47.65 | 47.31 | -0.34 | 3.4 | 3.8 | 0.4 |
| T0907-D1 | T0907TS479_1-D1 | T0907TS017_1-D1 | 80.17 | 73.28 | -6.89 | 3.08 | 3.02 | -0.06 |
| T0907-D2 | T0907TS479_1-D2 | T0907TS017_1-D2 | 65.94 | 61.88 | -4.06 | 2.72 | 2.63 | -0.09 |
| T0909-D1 | T0909TS251_5-D1 | T0909TS017_1-D1 | 41.07 | 41.07 | 0 | 3.32 | 2.59 | -0.73 |
| T0912-D2 | T0912TS220_1-D2 | T0912TS017_1-D2 | 62.95 | 65.36 | 2.41 | 2.38 | 1.65 | -0.73 |

Supplementary Table S6. Summary of ReFOLD performance on CASP12 TR domains. Mean GDT_TS of starting model = 66.93. ∑∆GDT_TS=-35.75. Mean MolProbity score of starting model = 2.0555. ∑∆MolProbity = 11.85. Data are from <http://www.predictioncenter.org/casp12/>.

|  | ModFOLD6_rank | | GDT-TS | | | MolProbity | | |
| --- | --- | --- | --- | --- | --- | --- | --- | --- |
| Target ID | Starting model | Refined model | Starting model | Refined model | Diff. | Starting model | Refined model | Diff. |
| TR520 | 0.4087 | 0.4098 | 79.13 | 79.05 | -0.08 | 1.85 | 2.44 | 0.59 |
| TR594 | 0.1042 | 0.11 | 55.34 | 58.43 | 3.09 | 2.95 | 2.29 | -0.66 |
| TR694 | 0.1825 | 0.1883 | 39.26 | 39.73 | 0.47 | 2.73 | 1.66 | -1.07 |
| TR862 | 0.2061 | 0.2126 | 58.6 | 58.6 | 0 | 2.13 | 2.09 | -0.04 |
| TR866-D1 | 0.2795 | 0.2867 | 79.57 | 77.16 | -2.41 | 1.55 | 6 | 4.45 |
| TR868-D1 | 0.3609 | 0.3708 | 80.95 | 79.29 | -1.66 | 0.5 | 1.58 | 1.08 |
| TR869 | 0.3407 | 0.3489 | 38.94 | 38.7 | -0.24 | 1.81 | 2.39 | 0.58 |
| TR870-D1 | 0.323 | 0.3642 | 42.43 | 44.27 | 1.84 | 3.44 | 2.24 | -1.2 |
| TR872 | 0.6081 | 0.6281 | 73.86 | 71.31 | -2.55 | 0.5 | 0.58 | 0.08 |
| TR874 | 0.4763 | 0.4788 | 52.7 | 52.93 | 0.23 | 1.25 | 1.89 | 0.64 |
| TR875 | 0.5239 | 0.5284 | 43.1 | 42.24 | -0.86 | 1.15 | 1.13 | -0.02 |
| TR876-D1 | 0.421 | 0.439 | 73.04 | 72.3 | -0.74 | 0.5 | 1.66 | 1.16 |
| TR877 | 0.6762 | 0.6805 | 70.25 | 69.19 | -1.06 | 1.29 | 2.48 | 1.19 |
| TR879 | 0.7004 | 0.7109 | 79.2 | 78.75 | -0.45 | 3.44 | 2.05 | -1.39 |
| TR881 | 0.5181 | 0.5226 | 69.43 | 69.55 | 0.12 | 2.68 | 3.03 | 0.35 |
| TR882 | 0.6089 | 0.6161 | 87.66 | 89.24 | 1.58 | 0.5 | 1.74 | 1.24 |
| TR884 | 0.5023 | 0.5023 | 65.84 | 65.84 | 0 | 2.44 | 2.44 | 0 |
| TR885-D1 | 0.5983 | 0.6114 | 92.07 | 87.74 | -4.33 | 0.88 | 1.69 | 0.81 |
| TR887-D9 | 0.4649 | 0.4756 | 68.17 | 64.13 | -4.04 | 1.38 | 1.99 | 0.61 |
| TR890 | 0.458 | 0.4653 | 45.48 | 43.75 | -1.73 | 2.01 | 1.66 | -0.35 |
| TR891 | 0.712 | 0.7191 | 91.3 | 90.62 | -0.68 | 1.4 | 2.53 | 1.13 |
| TR893 | 0.496 | 0.4988 | 87.28 | 87.13 | -0.15 | 1.51 | 1.23 | -0.28 |
| TR894 | 0.0818 | 0.085 | 74.54 | 67.13 | -7.41 | 2.51 | 1.41 | -1.1 |
| TR895 | 0.5488 | 0.5576 | 70.21 | 70.21 | 0 | 2.31 | 2.46 | 0.15 |
| TR896 | 0.0608 | 0.0632 | 60.76 | 57.85 | -2.91 | 2.14 | 1.7 | -0.44 |
| TR898 | 0.2328 | 0.252 | 37.03 | 39.86 | 2.83 | 0.66 | 1.97 | 1.31 |
| TR901 | 0.3851 | 0.3874 | 51.91 | 51.79 | -0.12 | 2.03 | 2.52 | 0.49 |
| TR905 | 0.45 | 0.4569 | 51.45 | 49.17 | -2.28 | 2.33 | 1.68 | -0.65 |
| TR909 | 0.4131 | 0.429 | 60.13 | 57.13 | -3 | 3.3 | 1.73 | -1.57 |
| TR910 | 0.6797 | 0.6825 | 85.67 | 85.37 | -0.3 | 0.75 | 0.73 | -0.02 |
| TR912 | 0.3081 | 0.3113 | 63.53 | 64.37 | 0.84 | 1.52 | 2.37 | 0.85 |
| TR913 | 0.5177 | 0.5207 | 66.2 | 63.91 | -2.29 | 1.32 | 2.25 | 0.93 |
| TR917 | 0.7115 | 0.7139 | 85.68 | 85.1 | -0.58 | 1.36 | 1.06 | -0.3 |
| TR920 | 0.1381 | 0.1425 | 79.68 | 75.23 | -4.45 | 1.57 | 2.06 | 0.49 |
| TR921 | 0.6309 | 0.6317 | 69.02 | 69.38 | 0.36 | 1.61 | 1.84 | 0.23 |
| TR922-D1 | 0.5207 | 0.5358 | 89.52 | 89.92 | 0.4 | 0.85 | 1.82 | 0.97 |
| TR928 | 0.5053 | 0.528 | 63.27 | 62.17 | -1.1 | 3.56 | 3.71 | 0.15 |
| TR942 | 0.3658 | 0.3766 | 52.33 | 51.49 | -0.84 | 2.26 | 2.02 | -0.24 |
| TR944 | 0.6035 | 0.6039 | 74.11 | 74.01 | -0.1 | 1.75 | 2.52 | 0.77 |
| TR945 | 0.4206 | 0.4265 | 59.27 | 61.2 | 1.93 | 2.31 | 2.34 | 0.03 |
| TR947 | 0.4503 | 0.4585 | 66.43 | 63.86 | -2.57 | 0.86 | 1.51 | 0.65 |
| TR948 | 0.5477 | 0.5544 | 76.68 | 76.17 | -0.51 | 1.59 | 1.84 | 0.25 |
